# Chinese technical terminology extraction based on DC-value and information entropy

**DOI:** 10.1038/s41598-022-23209-6

**Published:** 2022-11-21

**Authors:** Zhang Liwei

**Affiliations:** School of Management and Engineering, Capital University of Economics and Business, Beijing, 100070 China

**Keywords:** Information technology, Scientific data

## Abstract

China's technology is developing rapidly, and the number of patent applications has surged. Therefore, there is an urgent need for technical managers and researchers that how to apply computer technology to conduct in-depth mining and analysis of lots of Chinese patent documents to efficiently use patent information, perform technological innovation and avoid R&D risks. Automatic term extraction is the basis of patent mining and analysis, but many existing approaches focus on extracting domain terms in English, which are difficult to extend to Chinese due to the distinctions between Chinese and English languages. At the same time, some common Chinese technical terminology extraction methods focus on the high-frequency characteristics, while technical domain correlation characteristic and the unithood feature of terminology are given less attention. Aiming at these problems, this paper proposes a Chinese technical terminology method based on DC-value and information entropy to achieve automatic extraction of technical terminology in Chinese patents. The empirical results show that the presented algorithm can effectively extract the technical terminology in Chinese patent literatures and has a better performance than the C-value method, the log-likelihood ratio method and the mutual information method, which has theoretical significance and practical application value.

## Introduction

Terminology refers to a vocabulary unit describing the knowledge system of the professional domain, which contains abundant professional domain knowledge^1^. Terminology epitomizes and loads the core knowledge of a certain technology domain, whose change reflects the development trend of the technology domain, to some extent^2^. Terminology plays an important role in aspects of machine translation, scientific writing, question answering systems, automatic abstracting, knowledge communication, etc. Thus, many countries attach great importance to the construction of terminology corpuses, such as the EURODICAUTOM of European Union, LEXIS of the Language Office of the Federal Republic of Germany, TEAM of Siemens, TERMDOK of Sweden, DANETERM of Copenhagen Business School, the TER MINUM terminology group of Canada, the ROSTERM terminology base of Russian and so on^3^. Currently, the terminology in many technology domains mainly rely on artificial construction^4^, which is not only time-consuming, but also has a large cost^5^. Therefore, how to automatically extract terminology has been a concern for a long time.

Patent literature is the carrier of science and technology(S&T) information, recording the process of human S&T development. As the world's largest technology information source, patents cover 90–95% of the world's S&T information^6^. Most of the new inventions, new technologies, new crafts, and new equipment of various countries in various periods are reflected in patent literatures^7^. Currently, China's enormous economic market has attracted the attention of relevant people, both domestically and abroad. At the same time, China's technology is developing rapidly, and the number of patent applications has surged, with the number of patent applications in 2019 and 2020 continuously ranked first in the world. Therefore, how to apply computer technology to conduct in-depth mining and analysis of massive Chinese patent literature to make full use of patent information, perform technological innovation and avoid R&D risks has attracted widespread attention. Automatic term extraction is the basis of patent mining and analysis, but many existing approaches focus on extracting domain terms in English and are difficult to extend to Chinese due to the distinctions between Chinese and English languages. At the same time, some common Chinese technical terminology extraction methods focus on high-frequency characteristics, while the technical domain correlation characteristic and the unithood feature of terminology receive less attention.

In response to the above problems, this paper takes Chinese patent literature as the research object and proposes a method of extracting technical terms that combines grammatical rules and statistical methods to effectively identify technical terms and improve the accuracy of term extraction. The remainder of this paper is organized as follows. In “Theoretical background”, we describe existing work on automatic term extraction and focus on the challenges posed by domain-specific and unithood characteristics. In “The difference between Chinese and English in the process of extracting technical terms”, the difference between Chinese and English in the process of extracting technical terms is analysed. In “Terminology and patent terminology”, we present some basic notions associated with terms and the features of patent terms. We develop our proposed methodology for term extraction from Chinese patent literature in “Terminology extraction method based on domain C-value and information entropy”. Experimental evaluations and performance comparisons are given in “Experiment and results”. Finally, “Conclusion” concludes the method proposed in the paper and discusses the areas of future research.

## Theoretical background

Identifying and extracting domain terms from patent literature is a challenging task, which is mainly reflected in two aspects: on one hand, the domain terms in the literature are very professional and rarely appear in the general thesaurus; on the other hand, the phenomena of term abbreviations, entity inclusion, and mutual reference in the literature are very common, and which puts forwards higher requirements for the correctness and completeness of term recognition. Automatic term extraction methods can be summarized into several categories: rule-based methods, statistics-based methods, machine learning-based methods, deep learning-based methods, semantic correlation-based methods, graph-based methods, etc.

Rule-based term extraction methods mainly consider the context of the terms, the internal components of the terms and other factors to identify terms, use grammatical rules, semantic rules, etc. to match in the corpus and output multicharacter units that meet the established rules as terms. The common term extraction models mainly focus on language features^8,9^, syntactic patterns^10–12^, and retrieval strategies^13^. The advantages of the method include being concise, intuitive, and having a strong expressive ability. The method can apply expert knowledge, and the accuracy is high when the prior knowledge can match the text. However, this method usually requires an expert knowledge base as a foundation, and whether building a knowledge base manually or automatically, it requires the intervention and supervision of domain experts. At the same time, terms in different fields have different characteristics in terms of word composition. To obtain a better extraction effect, the knowledge base must be continuously updated and adjusted. In view of the shortcomings of the methods, such as poor adaptability, excessive manual intervention, inability to identify unknown words, etc., the application of this method has great limitations in terminology extraction.

The term extraction methods based on statistics apply various statistical models to measure whether a word string is a term in the sense of probability. The term evaluation measures can be categorized as termhood features and unithood features^14^. The main parameters used to compute the termhood and unithood of the candidate terms are frequency^15^, TF*IDF^16^, C-value/NC-value^17,18^, Domain Component Feature Set (DCFS)^19^, hypothesis testing (z-test, t-test, chi-square test, etc.)^20,21^, likelihood ratio (LR)^22,23^, information entropy^24,25^, mutual information (MI)^26,27^, etc. The advantages of the methods are mainly manifested in the following aspects: they are easy to implement and require less manual intervention; they are adaptable and can be used in different technical fields; and the unknown words can be identified. The disadvantages are as follows: they are not sufficiently concise and intuitive; they are very dependent on the corpus, and there must be a sufficient corpus to obtain a more ideal result; the accuracy rate is not high, because many related words in the probabilistic sense are not terms; the low frequency terms cannot be identified; and due to the need to perform many calculations, it is easy to cause operational efficiency problems.

The methods based on machine learning refer to the extraction of terms through machine training text features and constructing models. This method can compensate for the shortcomings of other methods that cannot identify low-frequency terms, and use the data learning models to determine the possibility of whether the word string is a term. Common machine learning methods include the maximum entropy model^28^ and the conditional random field model^29–31^. However, the methods based on machine learning have high requirements on the scale and quality of the training corpus, and a large-scale manual annotation corpus is required as the training data. Moreover, the methods are not yet mature, and more attempts and verifications are needed. There is currently no targeted, complete, and large-scale annotated corpus in patent literature.

The term extraction methods based on deep learning primarily combine the latest deep learning technologies to automatically extract terminology. It is a special machine learning method based on representation learning of data^32^ that can solve the problem of manually selecting the best feature in the extracted terms. Related studies have applied the deep learning methods based on neural networks to term extraction, for example, combining SVM^33^, Markov decision process^34^, Bi-LSTM^35–37^, CNN^38,39^, etc., to conduct research in order to avoid manual feature extraction and other issues. However, the methods highly rely on a large-scale annotated corpus, and manual annotation of the corpus is time-consuming and labour-intensive.

Currently, some new methods have appeared in the field of automatic term extraction, such as the term extraction methods based on semantic correlation, the extraction methods based on graphs, and so on. The extraction methods based on semantic correlation mainly use the semantic relationship between phrases to improve the ranking of terms, and thereby increase the accuracy of term extraction. Lahbib et al.^40^ applied the idea of semantic correlation to the field of bilingual term extraction, and extracted the source-end terms specific to the field. Astrakhantsev et al.^41^ proposed the KeyConceptRelatedness (KCR) method, which applied key concepts in the field to measure the quality of candidate terms. Yu et al.^42^ presented CBDLP,a data leakage prevention model based on confidential terms and their context terms. The graph-based term extraction methods are inspired by the ranking method of web page importance in PageRank. Mihalcea et al.^43^ first applied PageRank to the field of natural language processing(NLP), and proposed a TextRank method to extract key words. Semantic Graph-Based Concept Extraction (SGCCE), a novel concept extraction method was proposed by Qiu et al.^44^. Khan et al.^45^ presented the Term Ranker method, constructed an undirected weighted graph and improved the score of low-frequency terms.

In summary, related methods based on rules, statistics, machine learning, deep learning, etc. have all been used for technical term extraction, and these methods have their own advantages and disadvantages. Based on the existing research, this paper extracts the part-of-speech rules and grammatical rules of the terms in accordance with the strong domain characteristics of patent terms and constructs a Chinese patent term extraction model based on DC-value and information entropy theory.

## The difference between Chinese and English in the process of extracting technical terms

The biggest difference between Chinese and English is that in English, a "word" is used as the unit, where a single word can express a precise meaning, while in Chinese, the unit is generally a "character", and current Chinese emphasizes that "two-syllable words dominate". That is, it is difficult for each individual character to express a complete meaning. At least two characters are combined to form a word that has an accurate meaning.

At the same time, each word in English is divided by "spaces". Therefore, when extracting English terms, it is easy to extract individual words, but when extracting Chinese terms, it is difficult to express a complete meaning for each individual character, so usually words composed of multiple characters are extracted. In addition, English belongs to inflectional language, while Chinese is an isolated language. Thus, there are the following differences between English and Chinese: ① There are relatively rich inflections in English, and the relationship between words is expressed through inflections. ② An inflectional morpheme can express several different grammatical meanings in English. ③ The word order is strict in Chinese. Due to the lack of morphological changes in isolated words, there is no morphological sign of what component a word belongs to in a sentence; it is completely determined according to the word order. ④ Function words are very important in Chinese. The relationship between words in isolated languages is often reflected by function words, an important grammatical means.

## Terminology and patent terminology

A. Basic principles of terminology structure
  Terms are a type of language representation of concepts in a certain technology domain, and may be words, phrases, letters or digital symbols. According to the structure of terms, they can be divided into simple terms and complex terms^46^. Among them, the simple terms are composed of only one word, for example, "communication" and "information"; while complex terms can be broken down into smaller units with an independent meaning, for example, "communication apparatus" is made up of "communication" and "apparatus".
B. Features of patent terminology
  Because patent literature belongs to S&T literature, the terms extracted from patent literature have general characteristics of S&T terminology. The characteristics are roughly summarized as follows^47^:
C. Existing headwords. There are a few basic terms frequently appearing in a certain technology domain, which are very important and may be headwords. Then you can find that in the domain, many complex terms consist of the headwords in nominal structure or predicate structure. For example, in the password domain, a term that often appears is the word "key", which could be seen as the headword, to constitute the nominal structure, such as "session key", "master key", etc.; or the predicate structure, such as "key management", "key update", etc. Thus,a large number of compound terms are formed. In this technology domain, the word "key" is a headword.
D. Existing nested relationship among terms. Some complex terms are iteratively combined by simple terms, so there is a nested relationship among terms. For example, the nested relationship among "symmetric cryptography algorithms", "cryptography algorithms", and "algorithms" can be seen.
E. Constituting connecting structure by symbols. Terms are composed of symbols ("/", "-", ".", "_", etc.), such as "MH/NI battery", "D-H key exchange protocol", etc.
F. Combining English words with Chinese words to construct terms. Many terms are composed of both Chinese words and English words together to form technical terms.
G. Greater difference in length. There are not only existing terms with 2 or 3 characters, such as "电池" and "电动机", but also existing terms with lengths greater than 6 or 10, such as "反应式步进电机" and "管式固体氧化物燃料电池".
H. Uneven distribution in different domain. Because of the great difference in technical content in different technology domains, terms are closely related to technology domains, namely, the terms frequently appear in a technology domain but rarely emerge in other technology domains.

Patents can be products, production methods, or technical schemes^48^. In addition to the general characteristics of S&T terminology, patent terminology also has its own uniqueness, which is roughly as follows:

- The vast majority of patent terminology expresses the specific entity of objects, components, and other objective existences. This type of terms must include nouns that act as headwords.
- There exist a few terms representing abstract concepts of crafts and methods. These terms are mainly composed of verbs, and a few nouns, for example, "weld", "extract", "forge", etc.
- A term with more characters is, generally speaking, the object mainly described by the patent literature. The type of terms represent the latest technology frontier and need to be given significant attention, such as "electronic control gasoline injection engine", "plug-in series hybrid electric vehicle" and so on.

## Terminology extraction method based on domain C-value and information entropy

A. Framework of terminology extraction

**Figure 1:**
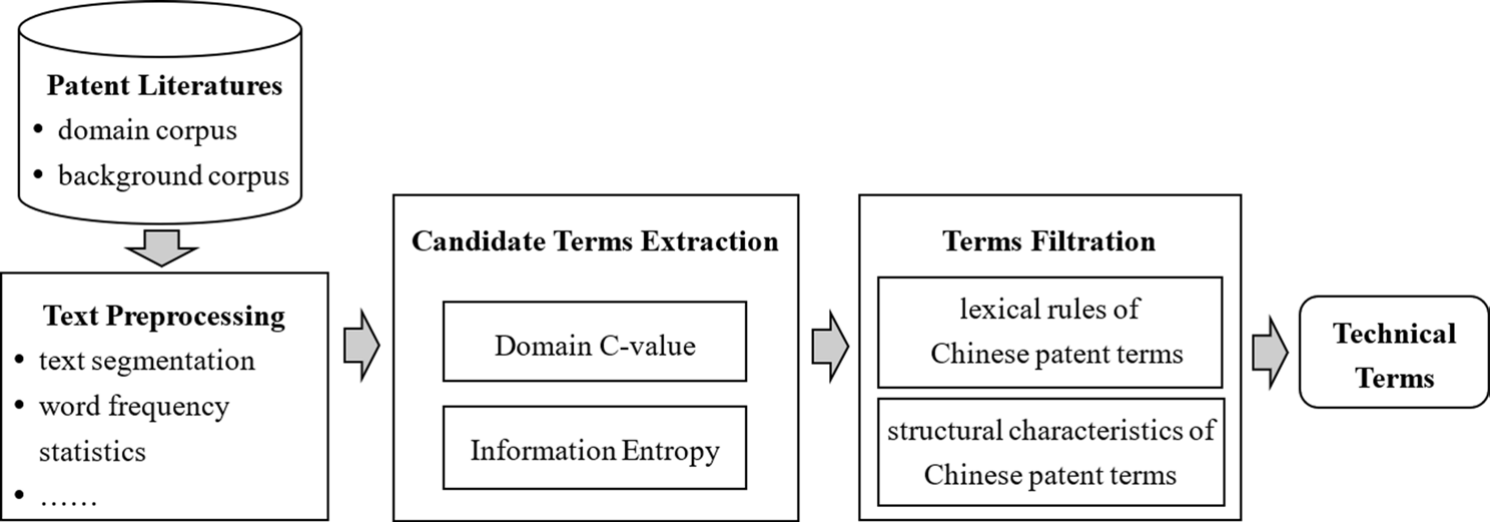
Technical terminology extraction framework of Chinese patents.

According to the characteristics of patent literature, the framework of technical terminology extraction is constructed, which is shown in Fig. 1.

The terminology extraction system is mainly composed of three parts: the text preprocessing module, the candidate terms extraction module and the terms filtration module.

B. Domain C-value (abbreviated as DC-value)

The C-value method is a type of hybrid terminology extraction method combining linguistic rules and statistical theory^17^. The calculation formula of the C-value is shown in Eq. (1)^49^:

1 $$C - value(s) = \left\{ {\begin{array}{*{20}l} {\log _{2} \left| s \right| \times f\left( s \right)\quad s{\mkern 1mu} {\text{ is}}{\mkern 1mu} {\text{not}}{\mkern 1mu} {\text{nested}}} \\ {} \\ {\log _{2} \left| s \right| \times \left( {f\left( s \right) - \frac{1}{{n\left( {b_{i} } \right)}}\sum\limits_{{i = 1}}^{{n\left( {b_{i} } \right)}} {f\left( {b_{i} } \right)} } \right)\quad s{\mkern 1mu} {\text{is}}{\mkern 1mu} {\text{nested}},} \\ \end{array} } \right.$$

where *s* represents a candidate term, |*s*| refers to the length of candidate term *s*, whose value is the number of characters included by *s*; *f*(*s*) represents the appearance frequency of *s*; *b*_*i*_ represents the candidate terms nesting *s*; and *n*(*b*_*i*_) is the number of *b*_*i*_.

However, the technical terms have the characteristics of domain correlation. The domain terms frequently appear or only appear in the texts belonging to a certain domain, while they rarely or never appear in other domains^50^. Therefore, the C-value method is optimized in this paper with the introduction of a background corpus^51^. Then the corpus is composed of two parts, the domain corpus and background corpus, based on which the domain C-value is constructed for the preliminary extraction of the candidate terms.

1. Domain C-value (DC-value)

DC-value is set as Eq. (2)

2 $$DC{ - }value = \left\{ {\begin{array}{*{20}l} \log_{2} \left| s \right| { \times sf\left( s \right) \times \frac{sf\left( s \right)}{{bf\left( s \right) + sf\left( s \right)}}} & \quad {s\,{\text{is}}\,{\text{not}}\,{\text{nested}}} \\ \log_{2} \left| s \right| \times \frac{{sf\left( s \right) - \frac{1}{{sc\left( {b_{i} } \right)}}\sum\limits_{i = 1}^{{sc\left( {b_{i} } \right)}} {sf\left( {b_{i} } \right)} }}{bf\left( s \right) + sf\left( s \right)} \times sf\left( s \right)& \quad {s\,{\text{is}}\,{\text{nested}}} {} & {} \end{array} } \right.$$

where *s* represents a candidate term; |*s*| refers to the length of *s*; *sf*(*s*) represents the frequency of *s* appearing in the domain corpus; *b*_*i*_ represents the extracted candidate terms nesting *s*; *sc*(*b*_*i*_) is the number of *b*_*i*_ in the domain corpus; and *bf*(*s*) represents the frequency of *s* appearing in the background corpus.

The extraction accuracy and performance of low-frequency words are effectively improved through the DC-value algorithm. However, the unithood feature is not considered. Aiming at this problem, the method of information entropy is introduced in subsequent research to ensure the integrity of the obtained terms.

III. Information entropy method

Information entropy in information theory represents the uncertainty of random variables. The more uncertain a random variable is, the larger its entropy value is. In the terminology extraction, the information entropy is mainly used to calculate the uncertainty of the boundaries of strings. The more uncertain the border of a string is, the larger the information entropy is. Then the string is more likely to be a complete term^52,53^.

The border uncertainty of strings is measured by computing the left and right information entropy of strings in this paper. For example, in the following paragraph "本发明提供一种转矩传感器以及动力转向装置。在具有一对解算器的转矩传感器中, 能够将上述两解算器的特性用作转矩传感器。" , the string “转矩传感器” has appeared a total of 3 times. Its left adjacent words successively are "种", "的" and "作", and its right adjacent words successively are "以", "中" and "。". In the entire corpus, the string "转矩传感器" appeares a total of 27 times. The number of different left adjacent words amounts to 15, and the number of different right adjacent words is 19. It can be seen that the left and right adjacent words are not fixed. Therefore, it can be inferred that "转矩传感器" is likely to be a complete phrase, or even a term.

In the study of whether the phrase of "转矩传感" is complete or not, the phrase "转矩传感" appeares 29 times. The different left adjacent words are 19, while the right ones are only 2. Thus, "转矩传感" is not suitable to be a complete phrase. Therefore, the uncertainty of this string collocation is estimated by calculating the information entropy of the string.Then, the formulas of the left and right information entropy are defined as follows^54^:

$$\begin{gathered} IE(s)_{L} = - \sum\limits_{l \in L} {p(ls|s)log_{2} (p(ls|s))} \hfill \\ IE(s)_{R} = - \sum\limits_{r \in R} {p(sr|s)log_{2} (p(sr|s))} , \hfill \\ \end{gathered}$$

where *s* is the candidate term, *IE*(*s*)_*L*_ and *IE*(*s*)_*R*_ respectively represent the left and right information entropy of *s*, *l* is the left adjacent word of *s*, *ls* is the phrase composed of *l* and *s*, *p*(*ls*|*s*) means the conditional probability that *l* is the left adjacent word of *s* in the case of the appearance of *s*, *r* is the right adjacent word of *s*, *sr* is the phrase consisting of *s* and *r*, and *p*(*sr*|*s*) means the conditional probability that *r* is the right adjacent word of *s* in the case of the appearance of *s*. The smaller *IE*(*s*)_*L*_ and *IE*(*s*)_*R*_ and the more fixed the left and right adjacent words are, then the less likely it is that *s* is an independent phrase.

To comprehensively evaluate the possibility of *s* standing alone as a phrase, the threshold values of the left and right information entropy are set to filter candidate strings that cannot stand alone as phrases^53^. The setting of the threshold is shown in the formula:

$$IE(s)_{R} \ge IE_{\min } {\text{ and }}IE(s)_{L} \ge IE_{\min } ,$$

where *IE*_*min*_ is a constant,it represents the minimum information entropy of word boundary and is set manually.

IV. Terminology filtration

In order to extract terms more fully and effectively, terminology filtering rules are set through a large amount of corpus analysis. The lexical rules and structural characteristics of Chinese patent terms are as follows:

- Location words, state words, interjections, and pronouns are not included in the terms;
- Terms should not begin with conjunctions, auxiliary words, or suffixes;
- Terms should not end with orientation words, auxiliary words, conjunctions, or prefixes;
- Nouns or verbs must be contained in terms;
- Adjectives and adverbs cannot stand alone as terms^55^;
- Focus on filtering symbols (such as "-", ". ", "_", "/", etc.);
- Focus on filtering the candidate terms containing English marks;
- The length of every term is less than 15;
- When a word does not appear in the stop word list and its part of speech is shown in Table 1, it needs to be filtered as a stop word.

**Table 1:** Part-of-speech tag table of special words.

Tag	Description	Tag	Description
t	Time word	q	Quantifier
m	Numeral	s	Location word
r	Pronoun	o	Onomatopoeia
p	Preposition	y	Modal
d	Adverb	z	State word
f	Position word	c	Conjunction

Part-of-speech tagging uses the Language Technology Platform (LTP) part-of-speech tag set.

## Experiment and results

A. Datasets construction

In this paper, the public service platform of Shanghai intellectual property (https://www.shanghaiip.cn/search/#/home) is applied as a patent retrieval database. The attributes of title, abstract, claims and international patent classification (IPC) are applied to retrieve the relevant patents, in which patents in the domain of information and communication are used as the domain dataset and patents in the domain of electric vehicles are used as the background dataset. We respectively selected 30,000 Chinese invention patents from the field of information and communication and the field of electric vehicles, where the retrieval time range was from 2010 to 2020. Then, a total of 60,000 items are used to construct a Chinese patent dataset. Among them, 20,000 items are respectively taken from the domain dataset and background dataset, and a total of 40,000 items are used as the training set. 10,000 items are separately taken from the domain dataset and the background dataset, and a total of 20,000 items are used as the test set.

To generate the initial candidate terms, we used the corresponding analysis tools and relational corpus-Chinese lexical analysers Language Technology Platform (LTP) and ACE RDC 2005 (Automatic Content Extraction Relation Detection and Characterization) Chinese corpus to perform data preprocessing for the domain dataset and background dataset. LTP provides a series of Chinese natural language processing tools that can be used to perform word segmentation, part-of-speech tagging, and syntactic analysis of Chinese text. The ACE RDC 2005 Chinese corpus contains three fields information——newswire, broadcast conversations and newspaper, and includes 85,575 relation instances, in which there are 8,469 positive instances. In this paper, LTP is applied to segment sentences into words and assign each word a POS tagging; ACE RDC 2005 is then used to merge synonyms or similar words. After the data preprocessing work has been completed, 50,129 initial candidate terms are obtained.

Table 2 shows the number of candidate terms after different selection and filtration steps. The results of each step are based on the results of the previous step.

**Table 2:** The selection and filtration of candidate terms.

Step	Term candidates after selection and filtration	Term candidates numbers
1	Initial candidate terms	50,129
2	Candidate terms after selection (DC-value + information entropy)	10,782
3	Candidate terms after filtration (structural characteristics + lexical rules)	3921

Finally, we successfully reduced the size of the candidate set from 50,129 to 3921.

B. Experimental results

**Figure 2:**
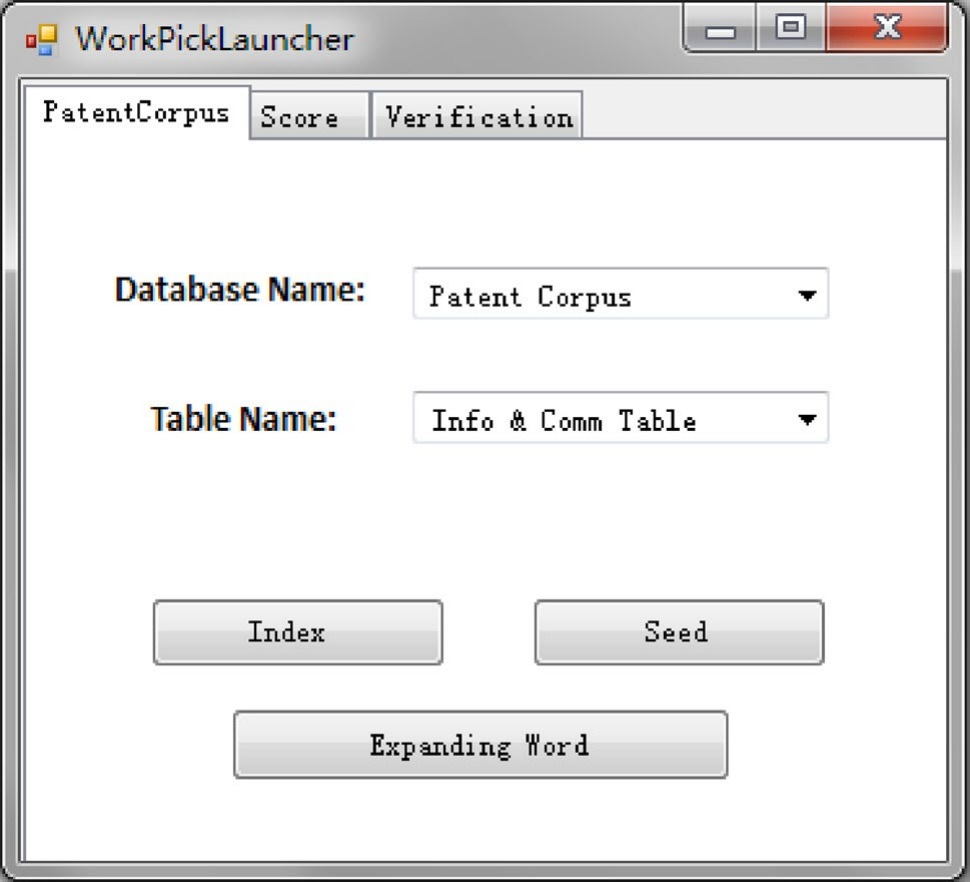
Patent corpus selection interface.

**Figure 3:**
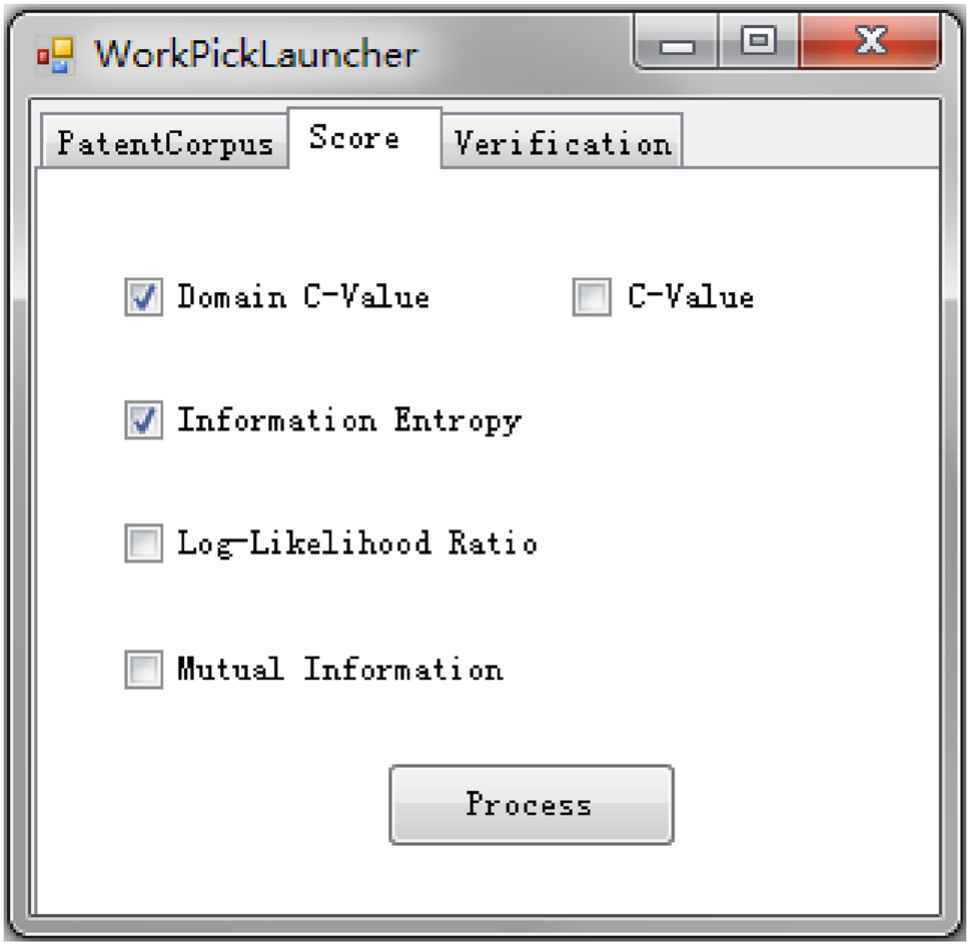
Extraction algorithm selection interface.

For ease of application, a technical terminology extraction tool has been developed. The tool interfaces are shown in Figs. 2 and 3.

The terms are then extracted by applying the extraction tools based on the methods of DC-value and information entropy algorithms. The results are shown in Table 3.

**Table 3:** Terminology extraction results.

Candidate terms	English translation	Frequency	Word segmentation	Part of speech	Terms?
多媒体子系统	Multimedia subsystem	7	多媒体 + 子系统	n + n	Yes
光突发交换	Optical burst switching	11	光 + 突发 + 交换	d + vi + v	Yes
光路交换	Optical circuit switching	13	光 + 路 + 交换	d + n + v	Yes
光分组交换	Optical packet switching	13	光 + 分组 + 交换	d + vd + v	Yes
偏振模色散补偿	Polarization mode dispersion compensation	16	偏 + 振 + 模 + 色散 + 补偿	d + vg + ng + n + vn	Yes
链路	Link	28	链 + 路	ng + n	Yes
媒体接入控制	Media access control	17	媒体 + 接入 + 控制	n + vn + vn	Yes
突发光发射	Burst mode transmitter	21	突 + 发光 + 发射	d + vi + v	Yes
突发光接收	Burst mode receiver	21	突 + 发光 + 接收	d + vi + v	Yes
无线资源调度	Radio resources Scheduling	23	无线 + 资源 + 调度	b + n + vn	Yes
无线资源管理	Radio resources management	23	无线 + 资源 + 管理	b + n + vn	Yes
正交频分复用	Orthogonal frequency Division multiplexing	21	正 + 交 + 频 + 分 + 复用	d + v + ag + v + vn	Yes
自动交换光网络	Automatically switched optical network	25	自动 + 交换 + 光 + 网络	d + v + d + n	Yes
多粒度光交换	Multi-granularity Optical switching	23	多 + 粒度 + 光 + 交换	m + n + d + v	Yes
多用户	Multiuser	25	多 + 用户	m + n	No
多粒度	Multi-granularity	27	多 + 粒度	m + n	No
多粒度光	Multi-granularity optical	25	多 + 粒度 + 光	m + n + n	No

III. Result analysis

Generally, two indicators, P (precision) and R (recall rate), are used to evaluate the effect of the term extraction. However, in a corpus that has not all been manually tagged, it is difficult to determine the total number of terms it contains. Therefore, an alternative method is adopted, that is, P is expressed as a percentage of the number of terms correctly extracted to the total number of terms extracted by the system; and R is expressed as the percentage of the number of terms correctly extracted by the system to the total number of manually tagged terms^48^.

Among them, the number of manually tagged terms were obtained by extracting 175 documents according to each IPC subcategory in the domain corpus. Finally, a total of 2625 documents were extracted, and a total of 559 manually tagged terms were obtained.

$${\text{P = }}\frac{{{\text{the}}\,{\text{number}}\,{\text{of}}\,{\text{correctly}}\,{\text{extracted}}\,{\text{terms}}}}{{{\text{the}}\,{\text{total}}\,{\text{number}}\,{\text{of}}\,{\text{extracted}}\,{\text{terms}}}} \times 100\% ,$$

$${\text{R = }}\frac{{{\text{the}}\,{\text{number}}\,{\text{of}}\,{\text{correctly}}\,{\text{extracted}}\,{\text{terms}}}}{{{\text{the}}\,{\text{total}}\,{\text{number}}\,{\text{of}}\,{\text{tagged}}\,{\text{terms}}}} \times 100\% .$$

To comprehensively evaluate the effect of the term extraction algorithm, the F-score evaluation index can be used, which is the harmonic mean of P and R, and the calculation formula is as follows^8^:

$${\text{F - Score = }}\frac{{{2} \times {\text{P}} \times {\text{R}}}}{{{\text{P}} + {\text{R}}}}.$$

In this paper, 60,000 patent documents in the domain of information and communication and in the domain of electric vehicles were processed through the extraction algorithms based on DC-value and information entropy. According to the extraction results of technical terminology, the P, R and F-Score indicators are calculated.

To truly reflect the performance of the term extraction method based on the DC-value and information entropy proposed in this paper, several current mainstream term extraction methods are used for a comparison. These methods include the C-value, likelihood ratio, and mutual information methods.

Part of the contrastive result and the performance comparison between the method proposed in this paper and the other three methods for the extraction of technical terminology are shown in Tables 4 and 5:

**Table 4:** Technical terminology extraction results of four methods.

Candidate terms	English translation	Terms or not?			
		DC-value + information entropy	C-value	Log-likelihood ratio	Mutual information
多媒体子系统	Multimedia subsystem	Yes	Yes	Yes	Yes
光突发交换	Optical Burst Switching	Yes	Yes	Yes	Yes
光路交换	Optical circuit switching	Yes	Yes	Yes	Yes
光分组交换	Optical packet switching	Yes	Yes	No	No
偏振模色散补偿	Polarization mode dispersion compensation	No	No	No	No
链路	Link	Yes	No	No	No
媒体接入控制	Media access control	Yes	Yes	Yes	Yes
突发光发射	Burst mode transmitter	Yes	Yes	No	Yes
突发光接收	Burst mode receiver	Yes	Yes	No	Yes
无线资源调度	Radio resources scheduling	Yes	Yes	Yes	Yes
无线资源管理	Radio resources management	Yes	Yes	Yes	Yes
正交频分复用	Orthogonal frequency division multiplexing	No	Yes	No	No
自动交换光网络	Automatically switched optical network	Yes	Yes	Yes	Yes

**Table 5:** Performance comparison among the methods.

Term extraction method	Precision (%)	Recall rate (%)	F-Score (%)
DC-value + information entropy	82.79	85.51	84.13
Information entropy	50.16	31.97	39.05
Log-likelihood ratio	78.16	81.32	79.71
Mutual information	80.27	79.30	79.78

Table 4 shows the extraction results of technical terminology by three different algorithms. Aiming at the same candidate terms, the judging result may be different.

Table 5 shows that the P, R and F-Score values of the terminology extraction algorithm based on the DC-value and Information Entropy are 82.79%, 85.51% and 84.13%, respectively, which is significantly better than the ones based on C-value, Log-likelihood estimation and mutual information methods. Therefore, the validity of the algorithm proposed in the paper is verified.

**Figure 4:**
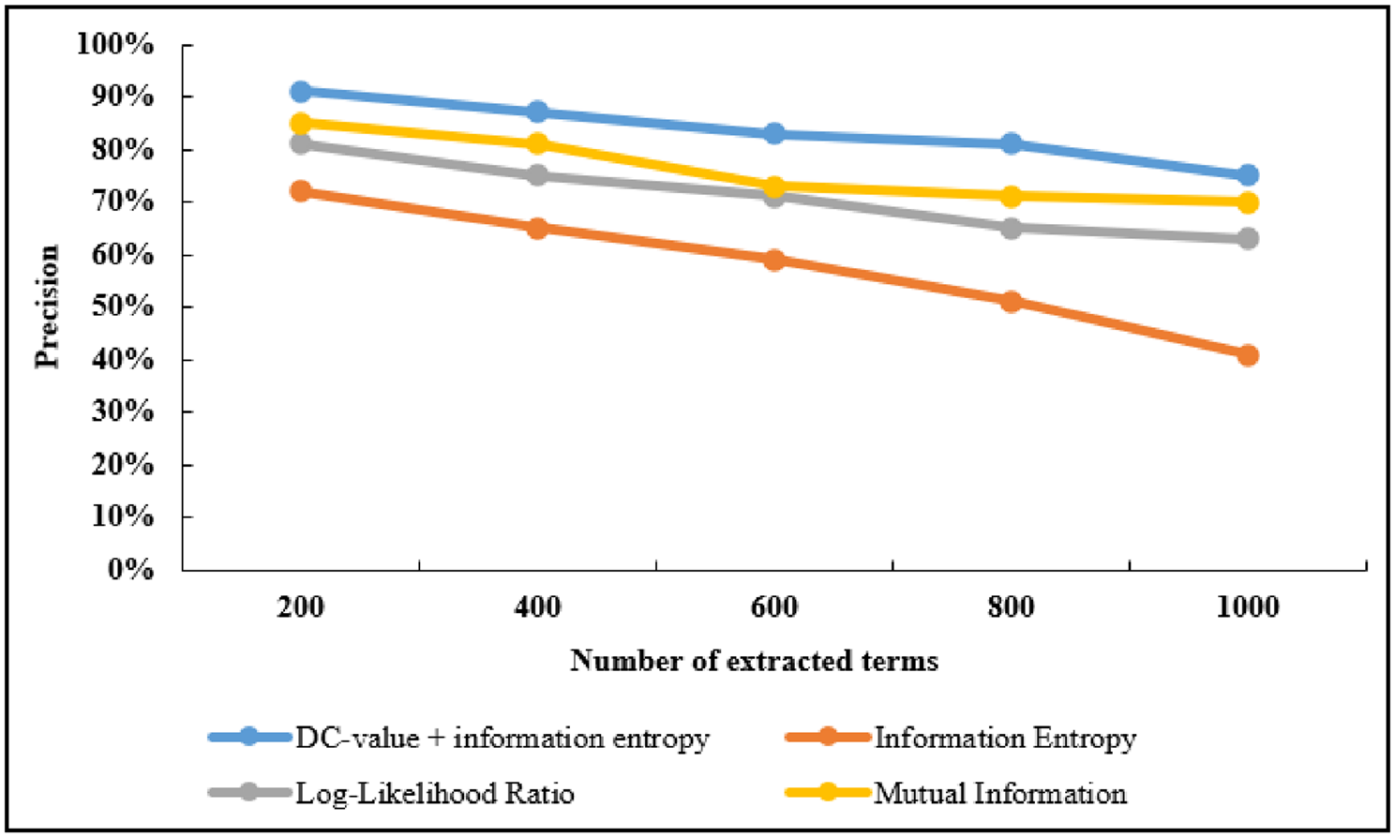
Precision comparison of extraction results.

**Figure 5:**
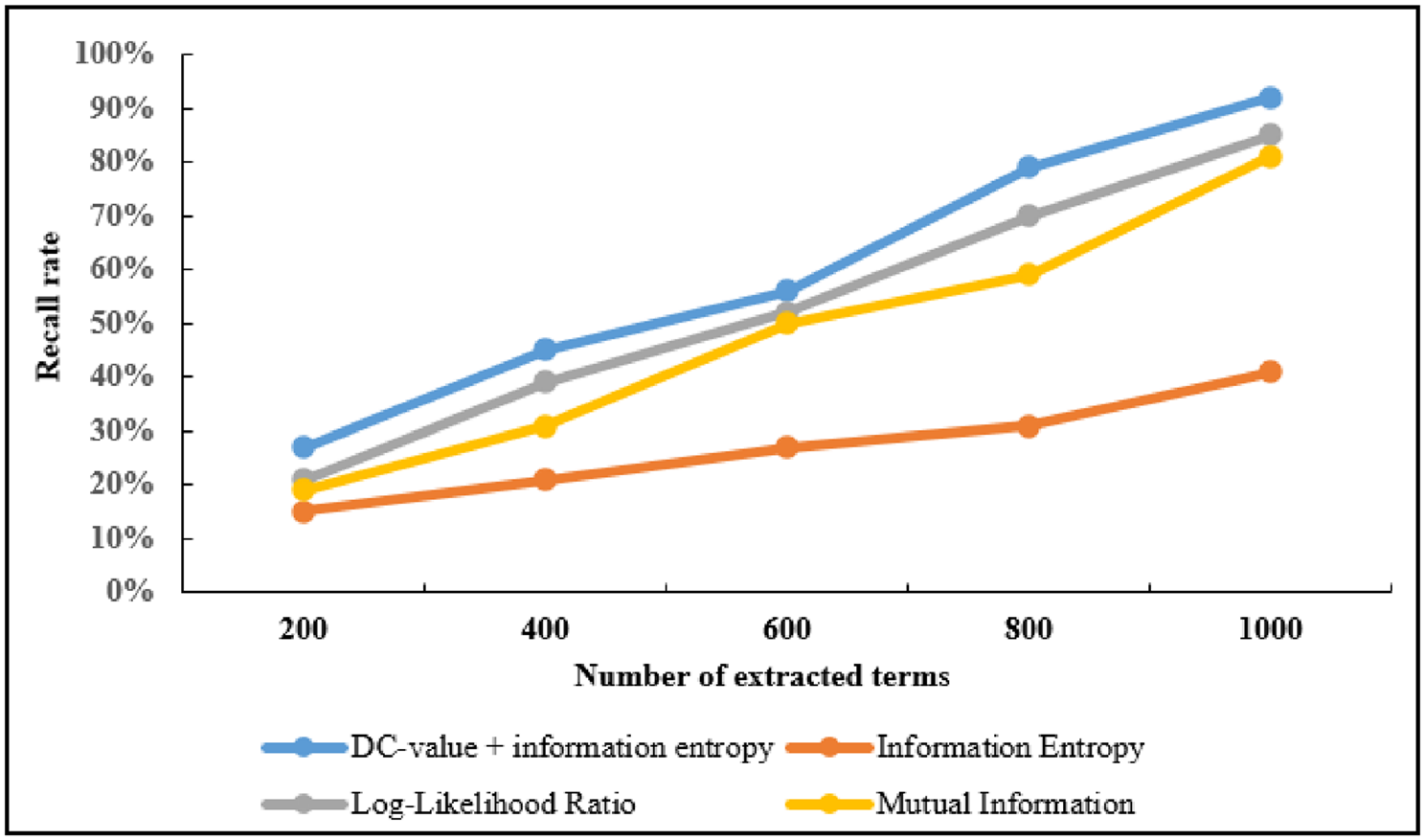
Recall rate comparison of extraction results.

At the same time, the experiment has compared the results of the four methods when 200, 400, 600, 800 and 1000 terms are extracted. The experimental results show as the number of extracted terms increases, the precision is decreasing, the recall rate is increasing, and F-Score is also increasing. The precision and recall rate of the first 1000 extracted terms among the four methods are compared, as shown in Figs. 4 and Figs. 5. In precision, the DC-value and information entropy method is 37%, 10% and 6% higher than the information entropy, log-likelihood ratio and mutual information methods, respectively. In recall rate, the DC-value and information entropy method is 49%, 7% and 11% higher than the information entropy, log-likelihood ratio and mutual information methods, respectively.

Through the analysis of experimental results, the method in this paper has been significantly improved compared with other methods.

1. Compared with the methods based on machine learning, the method in this paper does not require high-quality training corpus, and need not to spend a lot of time for corpus training. At the same time, through empirical studies in the fields of "biodegradable plastics", "carbon capture" and other fields, the effects are similar to the above, verifying that the method is applicable to various professional fields.
2. The extraction effect of combined terms and long terms is better. Due to the introduction of the background corpus, the setting of nested terms, the discrimination of term boundaries, etc., term recognition is more accurate. Through the analysis of the first 1000 candidate terms extracted, the extraction ratio of terms with 6 characters and above is higher than the extraction ratio of terms with less than 6 characters. For example, the term “生物降解专用树脂 (biodegradable special resin)” and the term "高强度导电聚乙烯醇 (high-strength conductive polyvinyl alcohol)" are both accurately extracted.

## Conclusion

Automatic term extraction is an important issue in natural language processing, and is the basis of patent mining and analysis. China currently attaches great importance to technological development, and China's patent applications have surged. Many (S&T) managers and researchers in different organizations urgently need conduct in-depth mining and analysis of massive Chinese patents in order to formulate accurate and effective technology research and development strategies. However, many existing approaches focus on extracting the domain terms in English and are difficult to extend to Chinese due to the distinctions between Chinese and English languages. Therefore, this paper proposed a Chinese patent term extraction method based on DC-value and information entropy to achieve automatic extraction of technical terms in Chinese patents.

Based on the traditional C-value method, this paper constructs the DC-value method to measure the termhood of terms. According to the characteristics of the terms, the relationship between terms and the context of terms is considered, and the left and right information entropy are used to calculate the boundary uncertainty of the strings. Through the above work, the selection of technical terms is completed according to the features of termhood and unithood. In addition, through the analysis of the structural features and lexical rules of Chinese patent terms, the filtering of technical terms is completed. The experiments show that the method in this paper has achieved better extraction results.

To improve the speed and accuracy of the algorithm, in future work, we will introduce association rules into the term extraction research to calculate the relevance of words, construct the relational structure of words or phrases and obtain domain terms. By deeply exploring the technology of automatic machine learning semantic relations between terms, the effectiveness and intelligence of term extraction can be improved.
